# Effects of probiotic-supplemented milk replacer on growth, blood biochemistry, fermentation, digestibility, and carcass traits in lambs

**DOI:** 10.1016/j.vas.2024.100368

**Published:** 2024-06-06

**Authors:** SH Mousavi Esfiokhi, MA Norouzian, MR Sahl Abadi, MR Rezaei Ahvanooei

**Affiliations:** Department of Animals and Poultry Science, College of Aburaihan, University of Tehran, Tehran, Iran

**Keywords:** Milk replacer, Probiotic, Digestibility, Fermentation parameters, Infant lamb

## Abstract

The objective of this study was to investigate the effects of feeding cow's milk replacer and probiotic supplementation on growth performance, blood metabolites, ruminal fermentation parameters, and nutrient digestibility in male lambs, with ewe's milk serving as the control treatment. Eighteen male lambs with an average initial body weight of 5.0 ± 1.2 kg and age of 15 ± 4 days were randomly assigned to three experimental groups: ewe's milk, cow's milk replacer, and cow's milk replacer with probiotic supplementation. The experiment was conducted using a completely randomized design. Lambs fed ewe's milk showed significantly higher average daily gain (218.4 g/day) compared to those fed cow's milk replacer (183.7 g/day) or cow's milk replacer with probiotic (209.1 g/day). Similarly, dry matter intake was highest in the ewe's milk group (585.6 g/day) compared to the cow's milk replacer (435.9 g/day) and cow's milk replacer with probiotic (510.5 g/day) groups. Blood glucose levels were higher in the ewe's milk group (75.3 mg/dL) compared to the cow's milk replacer (70.3 mg/dL) and cow's milk replacer with probiotic (72.1 mg/dL) groups. Probiotic supplementation resulted in increased blood urea nitrogen (15.6 mg/dL) and total protein (7.3 g/dL) levels compared to the other groups. Furthermore, the ewe's milk group showed higher apparent dry matter (76.1%) and crude protein (68.5%) digestibility compared to the other treatments. The fecal score on day 30 was higher in the cow's milk replacer group (2.34) compared to the ewe's milk (1.24) and cow's milk replacer with probiotic (1.45) groups. There were no significant differences in the carcass traits of the experimental lambs. In conclusion, based on the results of this study, it seems that feeding cow's milk + probiotic as a replacement for ewe's milk did not significantly affect the performance, carcass traits, and blood and fermentation parameters in infant lambs.

## Highlights

• This study was to investigate the effects of replacing of ewe's milk with milk replacer on improving functional growth characteristics including dry matter intake, daily weight gain and feed conversion ratio, nutrient digestibility, diarrhea, and improving some blood components.
• Feeding cow's milk as milk replacer with or without probiotic did not improve performance, nutrients digestibility and carcass traits of infant lambs compared to ewe's milk.
• Feeding milk replacer+probiotic reduced stool consistency score.

## 1 Introduction

The sheep industry in Iran faces significant challenges, particularly in the early stages of lamb rearing. Notably, the mortality rate of lambs during the early weeks of life can be substantial, with reports suggesting that it can reach as high as 20% in certain regions of Iran (Almasi et al., 2017). This high mortality rate can be attributed to various factors, including starvation, mismothering, inadequate nutrition, and environmental stress (Ridler et al., 2022). Lambs that lack sufficient milk intake are frequently frail and undernourished, making them susceptible to various diseases and high mortality rates.

While ewe's milk is considered the ideal source of nutrition for newborn lambs, providing it can be a challenge for sheep producers. Ewe's milk contains the optimal balance of proteins, fats, vitamins, and minerals essential for lamb growth, health, and development (Balthazar et al., 2017). However, in some cases, ewes may not produce sufficient milk or may need to be milked to maintain their body condition, necessitating the use of milk replacers. Feeding lambs with milk replacer can provide them with a proper balance of fat and protein, essential oils, and prebiotics, which support efficient growth for healthy and energetic lambs, gut health, feed-to-gain ratio, and nutrient absorption. Milk replacer can also be used to prepare ewes for the subsequent gestation period by reducing lactation length, thereby decreasing the lambing interval. Moreover, feeding lambs with milk replacer can help reduce lamb mortality rates and provide tailored care to bonus, orphan, weak, or small lambs (Ocak and Can Kaya, 2013).

Cow's milk replacers have been widely used as an alternative to ewe's milk for lamb rearing. These replacers are designed to mimic the nutrient composition of ewe's milk, but significant differences exist between the composition of ewe's and cow's milk, which can potentially impact the growth and development of lambs, especially in their early stages (Viñas et al., 2014).

In addition to milk replacers, the incorporation of dietary supplements with beneficial properties, such as probiotics, has gained interest in animal production systems. Probiotics are live microorganisms that, when administered in adequate amounts, can confer health benefits to the host by modulating the gut microbiota and improving nutrient utilization (Riddle et al., 2010). Several studies have demonstrated that the use of probiotics can improve various ruminal parameters, such as decreased lactate production, improved pH stability, and increased bacterial viability (Haddad et al., 2005; Kumar et al., 1997). Additionally, numerous studies have shown that the use of bacterial probiotics in the diet of newborn lambs (Noori et al., 2016; Kawakami et al., 2010) and calves (Ghorbani et al., 2002; Beauchemin et al., 2003) can enhance daily weight gain and feed conversion ratio. However, Agarwal (2002) reported that the effects of bacterial probiotics on performance, health status, and blood parameters have been inconsistent, likely due to factors such as the type of probiotic and feed used, management practices, probiotic administration methods, and environmental conditions.

The objective of this study was to examine the effect of substituting ewe's milk with a cow's milk replacer and probiotic supplementation on functional growth characteristics, such as dry matter intake, daily weight gain, feed conversion ratio, nutrient digestibility, and diarrhea incidence, as well as blood parameters in male lambs.

## 2 Materials and methods

### 2.1 Animals, treatments, and feeding

**Fig. 1:**
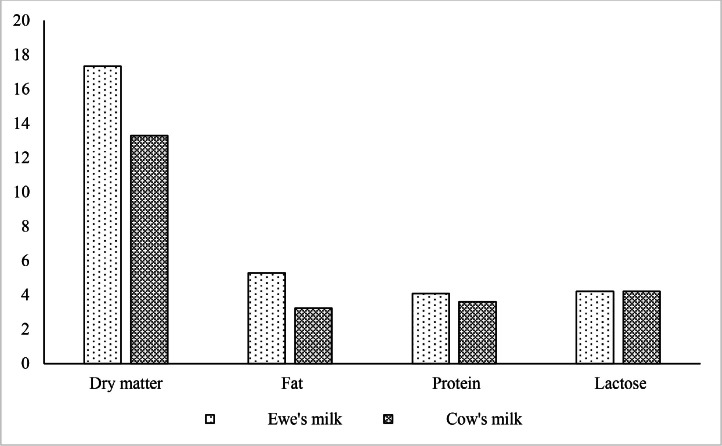
Nutrient compositions of milk and milk replacer (%).

Eighteen Zandi male lambs, with an initial body weight of 5 ± 1.2 kg and an age of 15 ± 4 days, were used in the study. The lambs were randomly allocated into 3 groups of 6 based on body weight, using a completely randomized design. The experimental treatments consisted of 1) control (ewe's milk), 2) cow's milk as a milk replacer, and 3) cow's milk supplemented with 2 gs of probiotic (Table 1, Fig 1).

**Table 1:** Ingredients and chemical composition of the basal total mixed ration fed to experimental lambs.

Ingredient	% DM	Composition	% DM
Alfalfa hay	5	Crude protein (% DM)	17.29
Barley	37	ME (Mcal/Kg DM)	2.85
corn	28	NDF (% DM)	20.2
Wheat bran	15	Calcium (% DM)	0.65
Soybean meal	14.1	Phosphorus (% DM)	0.38
Vitamin-Mineral mix	0.4		
Salt	0.3		
Di-calcium phosphate	0.3

The probiotic used was Protexin from Probiotics International Limited-United Kingdom, and the recommended feeding level by the manufacturer was 2 gs per day per lamb, mixed in the milk.

The milk replacer powder contains 96.91% dry matter (DM), 23.22% protein, and 13.20% fat. The daily milk replacer was dissolved in warm water (40 ± 1 °C). Lambs were given unrestricted access to milk or milk replacer via a pacifier glass, which was administered twice daily at 0800 and 1700 h. The milk equipment was cleaned with soapy bleach water and rinsed. Additionally, starter feed was introduced from the second week of the experiment, and feed was provided twice daily at 0800 and 1700 h in quantities that allowed for 10% refusal. The diets were manually mixed and weighed into each lamb's feed trough, and refusals were manually removed and weighed daily. The study was conducted for a duration of 90 days. Each lamb was housed in an individual pen with a cemented floor and provided with individual feeding and watering.

### 2.2 Measurements

At the beginning of the study, the lambs were individually weighed using a digital scale (100 kg capacity with a precision of 100 g), and subsequently weighed every two weeks. Daily feed intake was controlled with a 12-hour deprivation period. Blood samples were obtained from all lambs at the beginning of the experiment, as well as on days 45 and 90, via the jugular vein. The whole blood was centrifuged at 3000 × *g* for 15 min, and the resulting serum was extracted and frozen at −20 °C prior to analysis. Serum parameters including glucose, urea nitrogen, triglycerides, cholesterol, total protein, and albumin were assessed using commercial kits (Pars Azmoun, Tehran, Iran) and an auto-analyzer system (Model BT 3500, Spain). Two and a half milliliters of anticoagulated blood were utilized for complete blood cell count (CBC). The anticoagulated blood for red blood cell count and total leukocyte count was transferred to heparin-containing tubes, sent to the laboratory, and analyzed using microhematocrit, cyanmethemoglobin, and manual standard methods.

In the last three days of the trial, diets, refusals, and daily stools from the lambs were collected and combined to determine apparent in vivo digestibility using Acid Insoluble Ash (AIA) as an indigestible internal marker. Both the feed and fecal samples were ground to pass through a 1 mm plate and then stored in sealed plastic bags at room temperature. The nitrogen content of the feed and fecal samples was assessed using the Kjeldahl method, and the crude protein (CP) was calculated as *N* × 6.25 (AOAC, 1990). The neutral detergent fiber (NDF) in the feed and feces was determined using a fiber analyzer and the methods of Van Soest et al. (1991). Ash was determined through complete combustion in a muffle furnace at 450 °C for 8 h (AOAC, 1990). The ash samples were then boiled in 100 mL of 2 N HCl for 5 min and filtered through Whatman No. 541 filter paper in a vacuum system. Subsequently, the samples and filter paper were ashed for an additional 8 h. The dry matter and nutrient digestibilities were calculated using the following equations (Van Keulen & Young, 1977):

Drymatterdigestibility=100−[100(AIAinfeed/AIAinfeces)],

Digestibilityofnutrient=100−[(AIAinfeed/AIAinfeces)×(nutrientinfeces/nutrientinfeed)]×100.

To assess the ruminal fluid pH in the experimental lambs, sampling was conducted on the 90th day of the experiment, 2 h after feeding. This was achieved using an esophageal catheter with a plastic tube connected to a 50 ml syringe through the mouth. The pH of the samples was then determined using a portable digital pH meter (model 827 m) immediately after sampling and smoothing it with multilayer canvas fabric (Buchmin et al., 2003). The rumen liquid ammonia nitrogen was measured using the Conway (1950) titration method. To determine the volatile fatty acids, 10 ml of ruminal fluid was collected and filtered in a cap tube, after which a few drops of ordinary sulfuric acid were added. The test tube was then closed, centrifuged at 1000 rpm for 10 min, and the amount of volatile fatty acids was measured using the method of Croman et al. (1976).

The fecal consistency of the experimental lambs was evaluated on the 15th and 30th days of the experiment using a scoring system based on the table of Larson et al. (1977). The scoring system included four grades: Grade 1 (hard stool, loses a small amount of its original shape after falling and lying on the ground), Grade 2 (soft, unable to retain its original shape and somewhat smooth), Grade 3 (liquid and easily dispersed, about 6 mm thick), and Grade 4 (watery liquid and secretory consistency).

At the end of the feeding period, the lambs were slaughtered following a 12-hour feed removal, in accordance with the standard slaughter protocol at the experimental abattoir of the college of agriculture farm. Immediately after slaughtering, the carcass weight, tail, visceral fat, subcutaneous fat, liver, kidney, heart, spleen, testis, and the full and empty gastrointestinal tract were measured using a digital scale, with a precision of 5 gs.

### 2.3 Statistical analysis

The statistical analysis of the data was carried out using the GLM procedure in SAS (2004). Statistical analysis of the data for repeated measures (performance and blood parameters) were conducted using the MIXED procedure of SAS (2004). To detect statistical significance between treatments, Duncan's multiple range test was employed, with a significance level of 0.05.

## 3 Results

The findings regarding dry matter intake (DMI), daily weight gain, final live weight, and feed conversion ratio (FCR) are detailed in Table 2. The average daily gain (ADG) was significantly higher (*P* < 0.05) in lambs fed ewe's milk (218.4 g/day) compared to those fed cow's milk replacer (183.7 g/day) or cow's milk replacer with probiotic supplementation (209.1 g/day). Similarly, dry matter intake (DMI) was highest in the ewe's milk group (585.6 g/day), followed by the cow's milk replacer with probiotic (510.5 g/day) and cow's milk replacer (435.9 g/day) groups. The feed conversion ratio (FCR) was more favorable in the ewe's milk group (2.68) compared to the cow's milk replacer (2.37) and cow's milk replacer with probiotic (2.44) groups.

**Table 2:** The effect of different groups on feed intake and performance of experimental lambs.

Measurement	Ewe's milk	Cow's milk	Cow's milk+ probiotic	SEM	P-value	
					Treatment	Time
Initial body weight (kg)	5.12	5.28	5.05	1.19	0.84	–
Final body weight (kg)	21.50	19.03	20.73	1.06	0.13	–
Average gain (g/day)	218.40^a^	183.67^b^	209.07^ab^	20.2	0.02	0.002
Average dry matter intake (g/day)	585.56^a^	435.92^b^	510.45^b^	62.5	0.01	<0.001
Feed conversion ratio (FCR)	2.68	2.38	2.45	0.16	0.09	<0.001

Means with different superscript letters in rows are significantly different (*P* < 0.05).

Table 3 present the results for blood metabolites. Lambs fed ewe's milk showed higher (*P* < 0.05) blood glucose levels (75.3 mg/dL) compared to those fed cow's milk replacer (70.3 mg/dL) and cow's milk replacer with probiotic (72.1 mg/dL). Probiotic supplementation resulted in increased blood urea nitrogen (BUN) levels (15.6 mg/dL) and total protein (7.3 g/dL) compared to the ewe's milk (BUN: 12.4 mg/dL, total protein: 6.8 g/dL) and cow's milk replacer (BUN: 11.9 mg/dL, total protein: 6.5 g/dL) groups.

**Table 3:** Effect of different treatment on blood metabolite of experimental lambs.

Parameters	Ewe's milk	Cow's milk	Cow's milk+ probiotic	SEM	p-value		
					Treatment		Time
Glucose (mg/dl)	75.33^a^	70.27^b^	72.12^b^	1.65	0.0213	<0.001	
Blood urea nitrogen (mg/dl)	14.37^b^	13.21^b^	15.56^a^	2.23	0.0543		<0.001
Triglycerides (mg/dl)	45.08^ab^	44.67^b^	46.13^a^	2.60	0.0421		0.33
Cholesterol (mg/dl)	55.30	50.33	53.34	2.52	0.4010		0.34
High density lipoproteins (mg/dl)	24.58	23.75	25.27	0.80	0.3554		0.14
Low density lipoproteins (mg/dl)	9.20	11.85	12.15	1.60	0.2912		0.52
Albumin (g/ dl)	4.40	3.25	4.05	0.64	0.6011		0.41
Total protein (g/dl)	6.59^b^	5.64^c^	7.34^a^	0.08	0.0201	0.32	

Means with different superscript letters in rows are significantly different (*P* < 0.05).

Table 4 shows the experimental blood cell count (CBC) test data for the lambs. The values of all the blood parameters were found to be within the normal reference range for lambs, and the results did not indicate any significant differences between the experimental groups.

**Table 4:** Effect of different treatment on blood hematological parameters of experimental lambs.

Measurement	Ewe's milk	Cow's milk	Cow's milk+ probiotic	SEM	P-value
Red blood cell (RBC; 10^6^/µl)	9.78	9.06	9.68	0.12	0.18
White blood cell (WBC; 10^3^/µl)	11.07	10.12	10.45	0.76	0.24
Lymphocyte (%)	40.0	38.56	39.45	2.24	0.44
Neutrophil (%)	48.23	43.12	45.12	3.54	0.51
Eosinophil (%)	0.54	0.34	0.38	0.16	0.32
Monocyte (%)	1.20	1.15	1.05	0.42	0.64
Basophil (%)	0.15	0.14	0.17	0.28	0.43
Hematocrit (%)	42.10	40.12	41.45	1.45	0.57

Means with different superscript letters in rows are significantly different (*P* < 0.05).

Table 5 shows that no significant effects were observed for the digestibility of ADF, NDF, and ether extract. However, the apparent dry matter digestibility was significantly higher (*P* < 0.05) in the ewe's milk group (76.1%) compared to the cow's milk replacer (69.3%) and cow's milk replacer with probiotic (71.5%) groups. Similarly, the apparent crude protein digestibility was highest in the ewe's milk group (68.5%), followed by the cow's milk replacer with probiotic (62.7%) and cow's milk replacer (59.8%) groups.

**Table 5:** Effect of different experimental groups on apparent nutrient digestibility in lambs (based on DM).

Measurement	Ewe's milk	Cow's milk	Cow's milk+ probiotic	SEM	P-value
Dry matter (%)	76.11^a^	74.33^b^	75.23^b^	0.64	0.02
Crude Protein (%)	68.45^a^	65.72^b^	67.12^ab^	1.38	0.01
Neutral Detergent fiber (%)	56.35	57.25	57.24	0.49	0.26
Acid Detergent fiber (%)	46.70	44.70	45.10	0.96	0.54
EE (%)	78.24	70.14	75.12	0.45	0.32

Means with different superscript letters in rows are significantly different (*P* < 0.05).

The results of the tested rumen fermentation parameters are detailed in Table 6. The average level of ruminal ammonia nitrogen was affected by the experimental treatments and was higher for lambs that were fed ewe's milk (*P* < 0.05). Nevertheless, there were no significant variances in ruminal pH and volatile fatty acids concentration among the treatments.

**Table 6:** The effect of different treatments on ruminal fluid fermentation parameters in experimental lambs.

Parameters	Ewe's milk	Cow's milk	Cow's milk+ probiotic	SEM	P-value
pH	5.98	5.52	5.65	0.25	0.47
Ammonia nitrogen (mg/dL)	8.93^a^	7.34^b^	8.23^b^	0.34	0.02
Total volatile fatty acids (mmol/L)	105.23	95.56	97.10	4.34	0.57

Means with different superscript letters in rows are significantly different (*P* < 0.05).

On day 30, the fecal score, an indicator of diarrhea incidence, was higher (*P* < 0.05) in the cow's milk replacer group (2.34) compared to the ewe's milk (1.24) and cow's milk replacer with probiotic (1.45) groups (Table 7). As indicated in Table 8, there were no significant differences in the carcass traits of the experimental lambs.

**Table 7:** The mean fecal score and body temperature of lambs during different experiment periods.

parameters	Ewe's milk	Cow's milk	Cow's milk+ probiotic	SEM	P-value
Day15					
faeces stability	1.66	2.16	2.07	0.55	0.24
Body temperature	39.24	39.12	39.34	0.11	0.14
Day 30					
faeces stability	1.24^b^	2.34^a^	1.45^ab^	0.56	0.02
Body temperature	39.56	39.34	39.10	0.45	0.63

Means with different superscript letters in rows are significantly different (*P* < 0.05).

**Table 8:** Carcass characteristics of lambs with different experimental treatments.

Parameter	Ewe's milk	Cow's milk	Cow's milk+ probiotic	SEM	P-value
Warm carcass weight (kg)	10.87	9.27	10.01	0.10	0.11
Dressing percentage (%)	51.23	48.06	49.45	0.45	0.32
Legs (% of carcass)	0.55	0.43	0.45	0.43	0.81
Fat tail (% of carcass)	1.79	1.52	1.67	0.40	0.36
Testis (%of carcass)	0.23	0.18	0.21	0.44	0.53
Liver (%of carcass)	0.20	0.16	0.18	0.83	0.91
Kidneys (%of carcass)	0.12	0.10	0.12	0.21	0.11
Heart (%of carcass)	0.14	0.10	0.11	0.64	0.30
Lungs (%of carcass)	0.17	0.15	0.17	0.43	0.54
Digestive system (%of carcass)	3.05	2.76	2.87	0.54	0.24
Skin (%of carcass)	1.60	1.10	1.40	0.24	0.43
Head (%of carcass)	1.65	1.13	1.24	0.46	0.23

## 4 Discussion

The feeding of milk replacer and probiotics in lamb rearing has been widely adopted due to their potential to reduce milk consumption, thereby minimizing the utilization of body reserves in ewes. However, conflicting results have been reported regarding the effect of milk replacer and probiotics on infant lambs. The present study revealed that lambs fed ewe's milk showed higher dry matter intake (DMI) and daily weight gain compared to those receiving cow's milk replacer or cow's milk supplemented with probiotics. These findings are consistent with studies by Musharraf et al. (2014), who demonstrated that dairy lambs showed greater weight gain when fed ewe's milk as opposed to milk substitutes and cow's milk. Conversely, Napolitano et al. (2002) reported no significant difference in the daily weight gain of lambs reared on ewe's milk compared to those fed with free milk replacer.

Regarding the effect of probiotics, the inclusion of the probiotic supplement in the current study did not significantly influence the daily weight gain or final live weight of lambs. This observation aligns with the findings of several studies (Saleem et al., 2017; Baranowski et al., 2007), which reported no significant effect of probiotics on the daily weight gain of lambs before and after weaning. However, other researchers have reported contrasting results. For instance, Agarwal et al. (2018) demonstrated that the incorporation of probiotic supplements in lamb nutrition enhanced daily weight gain, attributing this improvement to increased nutrient digestibility. Similarly, Jatkauskas, Vrotniakiene et al. (2010) reported that the supplementation of probiotics in the milk replacer of young calves increased their daily weight gain and dry matter intake. Seifzadeh et al. (2017) also documented that the use of the probiotic Protexin in Holstein young calves resulted in increased feed intake. Furthermore, Hasunuma et al. (2011) suggested that a potential rise in the production of propionic acid in the rumen, following alterations in rumen microorganisms, contributed to more favorable weight gain in animals supplemented with probiotics.

The digestibility of nutrients is indicative of the gastrointestinal characteristics and productivity of lambs. In the present study, lambs fed cow's milk replacer showed lower digestibility of dry matter and crude protein in comparison to those fed ewe's milk. This finding is in line with previous reports, which have suggested that the lower digestibility of crude protein in milk replacers is attributed to reduced fat and protein digestibility compared to ewe's milk (Sanz Sampelayo et al., 1997; Qarabash, 2009). Additionally, it has been shown that the inclusion of probiotics did not affect the digestibility of dietary nutrients, including dry matter, organic matter, ADF, NDF, and raw ash (Hossein Abadi et al., 2013; Timas et al., 2018).

In contrast to the findings of the current study, previous research has demonstrated that probiotic additives led to significant changes in the blood urea concentration of lambs (Antunovic et al., 2005). Additionally, Fayed et al. (2005) reported that probiotics enhance the performance of ruminants by decreasing blood urea levels and utilizing it efficiently in the rumen for ammonia production and microbial protein synthesis. The observed discrepancy between our results and those of other studies could potentially be attributed to factors such as the specific probiotic strain used, the dosage and duration of supplementation, the composition of the basal diet, or the physiological stage of the lambs. It is important to note that the efficacy of probiotics can vary depending on the specific strains and their mode of action, as well as the interactions with the host animal's gut microbiome and diet. Additionally, the age and physiological state of the animals may influence the response to probiotic supplementation.

The results of a study indicated that the administration of bacterial Protexin in the milk ingested by calves resulted in a significant increase in serum protein and albumin concentration, along with a significant reduction in serum glucose concentration compared to the control treatment (Alsayadi et al., 2014), which was in accordance with our results. However, Moslemipour et al. (2013) reported that the total plasma protein concentration of calves remained unchanged with the addition of probiotics and mixed symbiotic to colostrum and milk.

The ruminal pH and total volatile fatty acids produced in lambs fed with ewe's milk and milk replacer + probiotic did not show any significant differences, which is consistent with the findings of Qarabash (2009) in lambs and Abaadi et al. (2013) in calves. However, other studies have reported that the pH and total volatile fatty acids of ruminal fluid in fattening lambs and sheep increase as a result of probiotic supplementation (Diaz et al., 2018; Li et al., 2011; Chung et al., 2011; Xiao et al., 2016). This discrepancy could be attributed to several factors, including the stage of rumen development in the infant lambs used in this study, the specific probiotic strain and dosage employed, the composition of the basal diet (particularly the proportion of readily fermentable carbohydrates), the duration of probiotic supplementation, and individual variations in factors such as initial rumen microbiome composition, feed intake, and health status of the animals.

In contrast to our findings, previous research has shown that the addition of probiotics to milk replacer increased the level of ruminal ammonia nitrogen in both lambs (Chashnidel et al., 2018) and calves (Shahmoradi et al., 2011). Hart and Glimp (1991) suggested that the development of the rumen and the enhanced fermentation capacity of nitrogenous compounds by existing microorganisms contribute to the elevation of ammonia nitrogen concentration in the ruminal fluid.

The study was conducted on infant lambs with developing rumens, and the probiotics may not have been able to effectively establish and influence the ruminal microbiome and fermentation processes. Additionally, the specific probiotic strains used in this study might have had different modes of action or interactions with the existing rumen microorganisms, leading to variations in nitrogen metabolism and ammonia production. Furthermore, the fermentation capacity of the ruminal microbiome in these infant lambs might not have been sufficiently developed to exhibit enhanced fermentation of nitrogenous compounds, as suggested by Hart and Glimp (1991) for more mature ruminants.

The inclusion of probiotics in milk replacer resulted in a reduction in stool consistency score, which is in line with the findings of Bahari et al. (2019) in lambs and Galvão et al. (2005) in calves. It seems that the utilization of probiotics in milk replacer has promoted a balanced microbial environment in the gastrointestinal tract, thereby decreasing the consistency of feces and the occurrence of gastrointestinal diseases (Kong et al., 2011).

Currently, there is limited information available regarding the effect of feeding cow's milk replacer, with or without probiotic supplementation on blood hematology in growing lambs. However, the findings of other studies on calves and piglets are consistent with our results. Shim (2005) reported that white blood cells, neutrophils, monocytes, and lymphocytes were not affected by probiotics, multicellular probiotics, and synbiotics in dairy pigs. One possible explanation for these outcomes could be the good physical condition of the mothers, which rendered the probiotics insignificant in terms of the concentration of hematological factors (Mohammadi Roodposhti, 2012).

There is a lack of sufficient information regarding the effect of probiotic-supplemented milk replacer on carcass traits in young lambs. Similar to our results, Raghebian et al. (2017) found that the consumption of probiotic-fortified milk had no significant effect on carcass traits and lamb meat quality. However, Bahari et al. (2014) reported that the administration of 2 g of probiotics and 1.5 g of prebiotics enhanced the carcass performance traits of the experimental lambs. The lack of significant differences in carcass traits, such as carcass weight, organ weights, and fat depot weights, among the experimental lambs fed ewe's milk, cow's milk replacer, or cow's milk replacer supplemented with probiotics, suggests that the dietary treatments did not substantially influence carcass composition and yield characteristics. This observation could be attributed to factors such as developmental stage of the lambs, the duration of the feeding trial, the similar nutrient composition of the basal diets, and the specific probiotic strain and dosage used, which may not have been effective in modulating the metabolic pathways governing carcass traits

## 5 Conclusion

The findings of this study suggest that the inclusion of probiotics in cow's milk replacer did not significantly impact the growth performance, blood hematology, or carcass traits of lambs during the pre-weaning phase. While some variations were observed in certain parameters, such as average daily gain and feed conversion ratio, these differences were not substantial enough to warrant a widespread adoption of probiotic supplementation in milk replacers for young lambs.

## Ethical statement

The authors confirm that the ethical policies of the journal, as noted on the journal's author guidelines page, have been adhered to and the appropriate ethical review committee approval has been received. The authors confirm that they have followed EU standards for the protection of animals used for scientific purposes.

## CRediT authorship contribution statement

**SH Mousavi Esfiokhi:** Methodology, Data curation, Conceptualization. **MA Norouzian:** Writing – review & editing, Supervision, Project administration, Methodology. **MR Sahl Abadi:** Methodology, Investigation, Conceptualization. **MR Rezaei Ahvanooei:** Writing – original draft, Validation, Methodology.

## Declaration of competing interest

The authors declare that they have no known competing financial interests or personal relationships that could have appeared to influence the work reported in this paper.
